# Associations of ultra-processed food intake with maternal weight change and cardiometabolic health and infant growth

**DOI:** 10.1186/s12966-022-01298-w

**Published:** 2022-05-26

**Authors:** Jenna R. Cummings, Leah M. Lipsky, Carolina Schwedhelm, Aiyi Liu, Tonja R. Nansel

**Affiliations:** 1 Social and Behavioral Sciences Branch, Division of Population Health Research, Eunice Kennedy Shriver National Institute of Child Health and Human Development, 6710B Rockledge Drive, Bethesda, MD 20817 USA; 2 Molecular Epidemiology Research Group, Max-Delbrueck-Center for Molecular Medicine in the Helmholtz Association, Berlin, Germany; 3 Biostatistics and Bioinformatics Branch, Division of Population Health Research, Eunice Kennedy Shriver National Institute of Child Health and Human Development, 6710B Rockledge Drive, Bethesda, MD 20817 USA

**Keywords:** Ultra-processed food, Pregnancy, Postpartum, Maternal weight change, Cardiometabolic health, Infant weight-for-length

## Abstract

**Background:**

Excessive intake of ultra-processed foods, formulated from substances extracted from foods or derived from food constituents, may be a modifiable behavioral risk factor for adverse maternal and infant health outcomes. Prior work has predominately examined health correlates of maternal ultra-processed food intake in populations with substantially lower ultra-processed food intake compared to the US population. This longitudinal study investigated relations of ultra-processed food intake with maternal weight change and cardiometabolic health and infant growth in a US cohort.

**Methods:**

Mothers in the Pregnancy Eating Attributes Study were enrolled at ≤12 weeks gestation and completed multiple 24-Hour Dietary Recalls within six visit windows through one-year postpartum (458 mothers enrolled, 321 retained at one-year postpartum). The NOVA (not an acronym) system categorized food and underlying ingredient codes based on processing level. Maternal anthropometrics were measured throughout pregnancy and postpartum, and infant anthropometrics were measured at birth and ages 2 months, 6 months, and 1 year. Maternal cardiometabolic markers were analyzed from blood samples obtained during the second and third trimesters.

**Results:**

Holding covariates and total energy intake constant, a 1-SD greater percent energy intake from ultra-processed foods during pregnancy was associated with 31% higher odds of excessive gestational weight gain (*p* = .045, 95% CI [1.01, 1.70]), 0.68±0.29 mg/L higher c-reactive protein during pregnancy (*p* = .021, 95% CI [0.10, 1.26]), 6.7±3.4% greater gestational weight gain retained (*p* = .049, 95% CI [0.03, 13.30]), and 1.09±0.36 kg greater postpartum weight retention (*p* = .003, 95% CI [0.38, 1.80]). No other significant associations emerged.

**Conclusions:**

Ultra-processed food intake during pregnancy may be a modifiable behavioral risk factor for adverse maternal weight outcomes and inflammation. Randomized controlled trials are needed to test whether targeting ultra-processed food intake during pregnancy may support optimal maternal health.

**Trial registration:**

Clinicaltrials.gov. Registration ID – NCT02217462. Date of registration – August 13, 2014.

**Supplementary Information:**

The online version contains supplementary material available at 10.1186/s12966-022-01298-w.

## Background

Excessive intake of ultra-processed foods, including snacks, drinks, ready meals, and other products formulated from substances extracted from foods or derived from food constituents, is widespread [1] and may compromise maternal and infant health. Ultra-processed foods are formulations resulting from a series of industrial processes including whole food fractioning, chemical modification (e.g., hydrolysis), assembly (e.g., pre-frying), additions for palatability (e.g., colors, flavors, emulsifiers), and packaging with synthetic materials [1]. These foods comprise more than half of the total dietary energy consumed in the US and other high-income countries [1]. A systematic review indicated that, in non-pregnant adults, greater ultra-processed food intake was associated with higher risk of overweight and obesity, all-cause mortality, overall cardiovascular disease, and overall cancer in cross-sectional and longitudinal studies [2]. Given that excessive maternal weight change [3] and infant weight [4] increase risk of adverse maternal and infant health outcomes, investigating their associations with ultra-processed food intake may elucidate a modifiable behavioral risk factor.

Ultra-processed food intake during pregnancy and postpartum may increase maternal weight gain and cardiometabolic disease risk, and increase infant weight through the prenatal environment and breastfeeding [5]. In Brazilian women, greater ultra-processed food intake during pregnancy was associated with greater gestational weight gain [6, 7], diabetes risk [6], and pro-inflammatory potential of diet [8]. Greater postpartum intake of ultra-processed foods was associated with lower concentrations of essential vitamins in breastmilk [9] and higher incidence of infant overweight [10]. Although the average energy intake from ultra-processed foods in the US is nearly double that of Brazil [1], only one small study (*n* = 45) has examined ultra-processed food intake during pregnancy in American women, finding associations with greater gestational weight gain and offspring adiposity at birth [11]. Further research is needed to understand relations of maternal ultra-processed food intake with maternal and infant weight and health outcomes in populations with high ultra-processed food intake.

The current longitudinal study examined relations of ultra-processed food intake during pregnancy and postpartum with maternal weight change and cardiometabolic health and infant growth in a US cohort. We hypothesized that greater ultra-processed food intake during pregnancy and postpartum would be associated with excessive maternal weight gain and retention, worse maternal cardiometabolic health, and greater infant growth.

## Methods

### Design, setting, participants, and procedures

The Pregnancy Eating Attributes Study (PEAS) examined dietary intake and weight change during pregnancy and postpartum in women from a metropolitan area in North Carolina, United States [12]. Eligibility criteria, including early pregnancy BMI (kg/m^2^) ≥18.5 and absence of pre-existing diabetes, any medical condition contraindicating study participation, participant-reported eating disorder, and use of medication that could affect diet or weight, were previously described in detail [12]. Mothers provided informed consent before enrollment, and visits occurred at <12 weeks (baseline/first trimester), 16-22 weeks (second trimester), and 28-32 weeks (third trimester) gestation and at 4-14 weeks (~2 months), 23-31 weeks (~6 months), and 50-58 weeks (~1 year) postpartum. At baseline, mothers were on average 30.5±4.7 years of age; 71.6% attained at least a bachelor’s degree; 71.4% were white; 47.8% were with normal weight (BMI ≥18.5, <25), 25.1% were with overweight (BMI ≥25, <30), and 27.1% were with obesity (BMI ≥30) (see Nansel et al., 2020 for other sociodemographic characteristics of the sample).

**Fig. 1:**
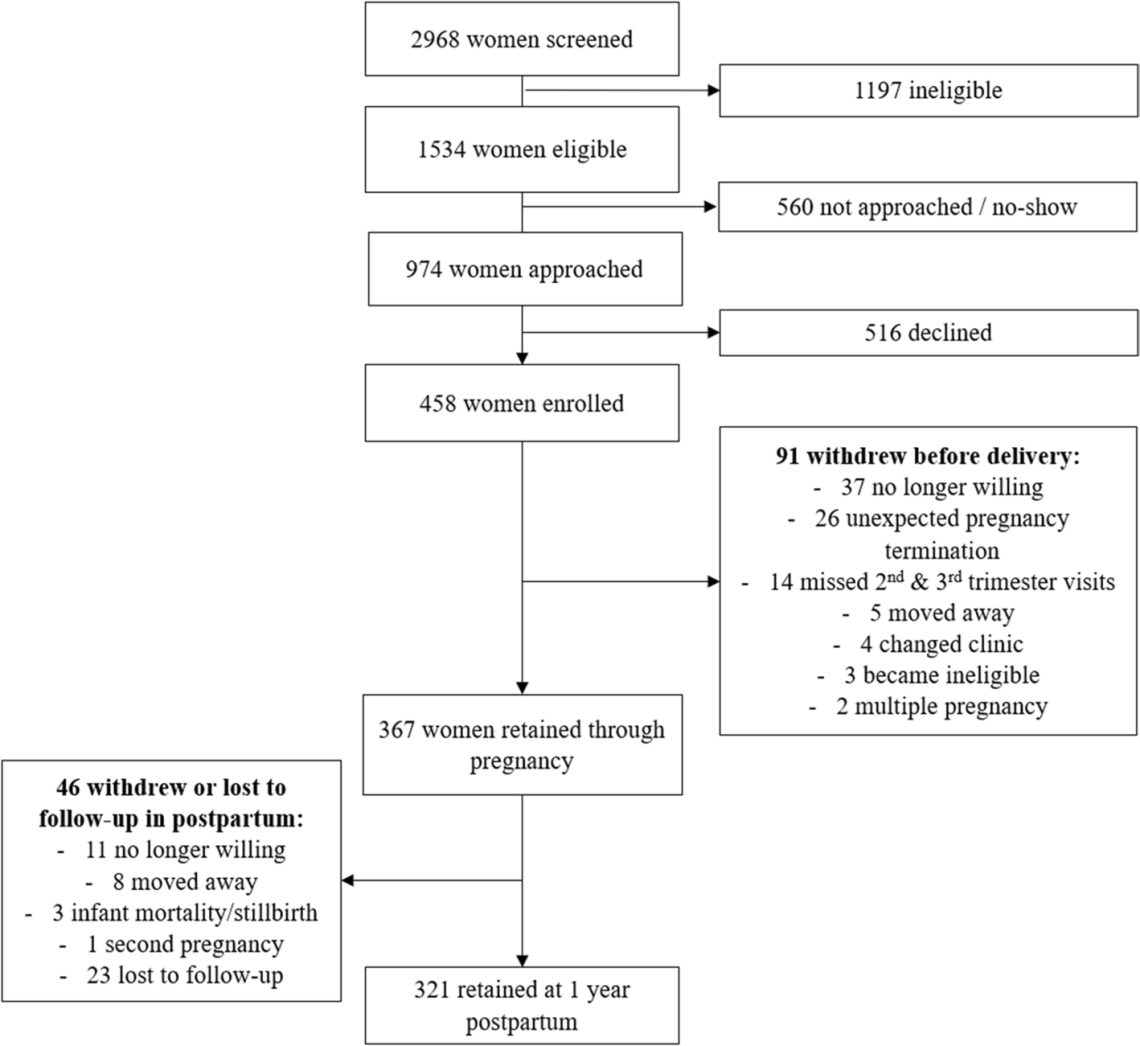
Flow Diagram of the number of mothers at each study stage

Procedures were approved by the University of North Carolina Institutional Review Board (study #18-2030) and were in accordance with the ethical standards of the Helsinki Declaration of 1975 as revised in 1983. Recruitment began in November 2014 and completed in December 2016; data collection completed in June 2018. Figure 1 presents a flow diagram of the number of mothers at each study stage. Of 458 mothers enrolled, 367 and 321 were retained through delivery and one-year postpartum, respectively [13]. The predeclared endpoint of PEAS was to examine the role of reward-related eating in maternal diet and weight change during pregnancy and postpartum (https://clinicaltrials.gov/ct2/show/NCT02217462). The present study was a secondary data analysis using all available data for variables of interest; since these analyses were not predeclared, they are considered exploratory.

### Independent variables

#### Dietary intake assessment

Mothers were asked to complete the well-validated Automated Self-Administered 24-Hour (ASA24) dietary recall [14] once within each visit window, indicating all foods consumed in the past 24 hours, including details on food preparation, brands, portion size, and additions. The ASA24 program assigns food codes from the US Department of Agriculture Food and Nutrient Database for Dietary Studies (FNDDS) and provides nutrition information including kilocalories [14]. Research staff at the University of North Carolina Nutrition and Obesity Research Core corrected implausible and missing food codes and nutrition information. Records (1.9%) with implausible daily energy intake (< 600 kcal/day), based on established cutoffs adjusted for increased energy requirements of pregnancy, were excluded from analysis resulting in exclusion of one participant during pregnancy and one participant during postpartum [15, 16].

To assess maternal ultra-processed food intake, food codes were categorized according to the NOVA (not an acronym) classification system, which is a set of descriptive criteria developed by an academic group at the University of São Paulo [1]. Standardized Stata (College Station, TX) code for applying NOVA was used [17], which is available upon request from the University of São Paulo group. Ultra-processed food categorization according to NOVA has demonstrated convergent validity with calculations of added sugars and macro- and micro-nutrients [1]. There are four categories:

- group 1 – unprocessed or minimally processed foods, which are foods in their natural form or altered by industrial processes such as removal of inedible or unwanted parts, drying, crushing, grinding, fractioning, roasting, boiling, pasteurization, refrigeration, freezing, placing in containers, or vacuum packaging (e.g., fresh, squeezed, chilled, frozen, or dried fruits and vegetables; fresh, chilled, and frozen meat, poultry, fish, and seafood, whole or in the form of steaks, fillets, and other cuts; fresh or pasteurized milk; pasta, couscous, and polenta made with flours, flakes, or grits and water),
- group 2 – processed culinary ingredients, which are substances obtained directly from group 1 foods or from nature by industrial processes such as pressing, centrifuging, refining, extracting, or mining (e.g., vegetable oils crushed from seeds, nuts, or fruits; butter and lard obtained from milk and pork; sugar and molasses obtained from cane or beet),
- group 3 – processed foods, which are products made by adding group 2 ingredients to group 1 foods, using preservation methods such as canning or bottling and industrial processes such as non-alcoholic fermentation (e.g., freshly made unpackaged breads and cheeses; fruit preserves; salted or sugared nuts and seeds),
- group 4 – ultra-processed foods (e.g., ‘instant’ foods; ready-to-heat pre-prepared pies, pasta, and pizza dishes; mass-produced packaged breads; reconstituted meats; sweet or savory packaged snacks; confectionery desserts; sweetened drinks).

For food codes indicating a homemade recipe, underlying ingredient codes and correspondent nutrition information were obtained from the FNDDS and categorized according to NOVA [17]. See Additional file 1 for the 15 most-frequently reported foods from the ultra-processed food category in the current study sample and see Monteiro et al. [1] for full definitions and lists of examples of foods assigned to each NOVA category.

Given that there is little change in dietary intake across pregnancy trimesters [18–20], total daily energy intake (kcal/day) during pregnancy and postpartum were calculated by averaging total daily energy intake across all ASA24 dietary recalls collected during pregnancy and across all ASA24 dietary recalls collected during postpartum, respectively. Percent daily energy intake from ultra-processed foods during pregnancy and postpartum were calculated by dividing the average total daily energy from ultra-processed foods by the average total daily energy intake [17]. The intent of using percent daily energy intake from ultra-processed foods was to reduce bias introduced by non-differential calorie misreporting from all foods [21].

### Dependent variables

#### Maternal anthropometrics

At baseline, maternal height was measured to the nearest 0.1 cm using a wall-mounted stadiometer. At each study visit, weight was measured to the nearest 0.1 kg using a standing scale, and skinfolds thickness was measured to the nearest 0.1 mm using skinfold calipers. Each anthropometric measurement was obtained twice; if the two measurements varied by more than 1 cm (height), 0.2 kg (weight), or 2 mm (skin folds), a third measure was taken. The mean of the two closest measurements was calculated. Early pregnancy BMI was calculated from baseline height and weight. Patient medical records indicated maternal weight at prenatal medical visits. Consistent with clinical practice and prior research [22, 23], gestational weight gain was calculated as the difference between baseline weight and the last prenatal medical visit weight [*M*(*SD*) = 0.35(0.75) weeks prior to delivery]. Gestational weight gain was categorized as inadequate, adequate, or excessive using 2009 Institute of Medicine guidelines [24], which indicate optimal range of weight gain according to pre-pregnancy BMI (here, early pregnancy BMI). Gestational weight gain was categorized because the direction of association between absolute gestational weight gain and health outcomes differs depending on these categories [24]. Gestational fat gain (kg) was calculated from weight and thigh skinfold change using an equation validated for pregnancy: 0.77*weight change + 0.07*thigh skinfold change – 6.13 [25]. Percent of gestational weight gain retained was calculated by multiplying 100 by the difference in weight from last prenatal medical visit to 1-year postpartum divided by gestational weight gain; percent of gestational weight gain retained (but no other maternal anthropometrics) was coded as missing for participants who had ≤2 kg gestational weight gain. Postpartum weight change (kg) was calculated by subtracting baseline weight from weight at 1-year postpartum.

#### Cardiometabolic markers

At the second trimester visit, mothers were instructed to fast for at least 8 hours before blood samples (40 ml) were collected; HDL (mg/dL), LDL (mg/dL), triglycerides (mg/dL), glucose (mg/dL), insulin (pmol/L), and c-peptide (nmol/L) were analyzed from the fasting second-trimester samples. At the third trimester visit, non-fasting blood samples (30 ml) were collected; Interleukin 6 (IL-6; pg/mL), tumor necrosis factor alpha (TNF-a; pg/mL), and c-reactive protein (CRP; mg/L) were analyzed from the non-fasting third-trimester samples. While natural changes in cardiometabolic markers occur during pregnancy, excessive elevations of cardiometabolic markers, particularly during the second and third trimesters, have been associated with adverse maternal and child outcomes [26, 27]. Samples were processed within 30-60 minutes after collection including ensuring proper distribution of anticoagulant, transferring to cryovials, and affixing barcode labels. Samples were stored in a freezer at -20°C for up to 5 days and then transferred on dry ice to a freezer at -80°C. Lipids and CRP were measured using standardized methods on a Roche COBAS 6000 (Roche Diagnostics, Indianapolis, MN) and IL-6 and TNF-a were measured using standard sandwich ELISAs (R&D Systems, Minneapolis, MN).

#### Infant anthropometrics

Patient medical records indicated infant weight (kg) and length (head to foot; cm) at birth. At 2 months, 6 months, and 1 year, infant weight was measured to the nearest 0.01 kg on an infant scale and length was measured to the nearest 0.1 cm using a recumbent infant board with a stadiometer. Each measure was obtained twice; if the two measurements varied by more than 0.2 kg (weight) or 1 cm (length), a third measure was taken. The mean of the two closest measurements was calculated. Infant weight-for-length z-scores were calculated based on US Centers for Disease Control and Prevention reference growth curves for infant age and sex [28].

### Covariates

The following covariates were considered because of their potential causal influence on both ultra-processed food intake and dependent variables: maternal age, low-intensity physical activity, moderate- and vigorous-intensity physical activity, and income-poverty ratio at baseline and smoking status and alcohol use during pregnancy. Patient medical records indicated maternal age (in years) at baseline and smoking status (1 = “Smoker During Pregnancy,” 2 = “Former Smoker,” 3 = “Never Smoker”) and alcohol use during pregnancy (0 = “No,” 1 = “Yes”). At baseline, mothers reported how often they typically engage in multiple physical activities (e.g., bicycling, dancing, tennis) via questions adapted for pregnant women in the National Health and Nutrition Examination Survey [29]; the intensity of the activities was coded as low, moderate, or vigorous based on standard MET intensities [30], and low-intensity and moderate- and vigorous-intensity physical activity variables were calculated by summing the number of times per week mothers engaged in respective activities. Mothers reported their total annual household income, and income-poverty ratio was calculated by dividing this by the US Census Bureau 2016 poverty thresholds accounting for household size and number of children [31].

### Statistical analysis

Except for the cardiometabolic markers and weight change variables, which have clinically interpretable units, continuous variables were z-scored for ease of interpretation. Linear regressions estimated relations of maternal ultra-processed food intake during pregnancy and postpartum with maternal weight change and cardiometabolic markers. Logistic regression examined relations with gestational weight gain (adequate used as reference category). Model 1 included percent energy intake from ultra-processed foods as the only independent variable; Model 2 added covariates and total energy intake.

To determine which covariates to include in Model 2, bivariate associations of potential covariates with independent and dependent variables (uncorrected for multiple comparisons) were examined (see Additional files 2 and 3). Variables that were significantly associated (*p* < .05) with both independent and dependent variables were included as covariates. While total energy intake was only modestly associated with ultra-processed food intake during pregnancy and postpartum, it was included as a covariate to examine the independent associations of total energy intake and ultra-processed food intake with dependent variables. In addition, early pregnancy BMI was included as a covariate (a) in the gestational fat gain analysis because gestational fat gain was inversely correlated with early pregnancy BMI and adverse cardiometabolic health during pregnancy and (b) in the CRP analysis because prior work indicates adiposity (as proxied by BMI) is a major determinant of CRP independent of dietary intake [32].

To examine relations of maternal ultra-processed food intake during pregnancy and postpartum with infant weight-for-length, multilevel growth modeling nesting repeated weight-for-length measures within infants was conducted with time (coded as 0, 1/6, 1/2, and 1 year) entered at Level 1. Time was centered such that the model’s intercept represented infant weight-for-length at birth and the model’s slope represented infant weight-for-length trajectory from birth to 1 year. Random effects for intercept and slope were included because they improved model fit as indicated by a significantly smaller -2LL (log-likelihood). In Model 1, intercept was predicted by maternal ultra-processed food intake during pregnancy only and slope was predicted by maternal ultra-processed food intake during pregnancy and postpartum (entered at Level 2). The system of equations was as follows, wherein *t* = time and *i* = individual:

- Level 1: Infant Weight-for-length_ti_ = *β*_0i_ + *β*_1i_(Time_ti_)+ *ɛ*_ti_
- Level 2: *β*_0i_ = *γ*_*00*_*+ γ*_01_(%Energy Intake from Ultra-Processed Foods During Pregnancy_0i_) + *u*_*0i*_
  *β*_1i_ = *γ*_*10*_*+ γ*_11_(%Energy Intake from Ultra-Processed Foods During Pregnancy_1i_) *+ γ*_12_(%Energy Intake from Ultra-Processed Foods During Postpartum_1i_) + *u*_*1i*_

In Model 2, intercept was additionally predicted by maternal total energy intake during pregnancy, and slope was predicted by maternal total energy intake during pregnancy and postpartum (entered at Level 2). Analyses were conducted in SAS 9.4 (Cary, NC) using maximum likelihood estimation/restricted maximum likelihood estimation (REML) to account for missing data. Significance was set at *p* < .05.

## Results

Univariate statistics for variables of interest are presented in Table 1. On average, participants consumed 52.6±15.1% and 50.6±16.6% of energy intake from ultra-processed foods during pregnancy and postpartum, respectively.

**Table 1:** Univariate statistics for variables of interest

	*n*	
**Maternal**		
Age at Baseline, years (*M*, *SD*)	458	30.46 (4.74)
Week of Gestation at Baseline (M, SD)	458	9.82 (1.75)
Smoking Status (*n*, *%*)	291	
Smoker During Pregnancy		7 (2.4%)
Former Smoker		51 (17.5%)
Never Smoker		233 (80.1%)
Alcohol Use During Pregnancy (*n*, *%*)	290	
No		164 (56.6%)
Yes		126 (43.5%)
Low-Intensity Physical Activity, times/week (*M*, *SD*)	294	3.81 (4.19)
Moderate- and Vigorous-Intensity Physical Activity, times/week (*M*, *SD*)	294	1.07 (2.00)
Income-Poverty Ratio (*M*, *SD*)	364	3.84 (1.97)
Total Energy Intake During Pregnancy, kcal/day (*M*, *SD*)	365	2017.46 (678.74)
%Energy Intake from Ultra-Processed Food During Pregnancy (*M*, *SD*)	365	52.58 (15.12)
Total Energy Intake During Postpartum, kcal/day (*M*, *SD*)	266	1980.22 (655.07)
%Energy Intake from Ultra-Processed Food During Postpartum (*M*, *SD*)	266	50.57 (16.62)
Early Pregnancy BMI, kg/m^2^ (*M*, *SD*)	458	27.19 (6.94)
Gestational Weight Gain (*n*, *%*)	367	
Inadequate		70 (19.1%)
Adequate		124 (33.8%)
Excessive		173 (47.1%)
Gestational Fat Gain, kg (*M*, *SD*)	355	0.48 (3.46)
%Gestational Weight Gain Retained (*M*, *SD*)	285	3.56 (51.82)
Postpartum Weight Change, kg (*M*, *SD*)	293	0.78 (5.37)
HDL During Second Trimester, mg/dL (*M*, *SD*)	360	73.43 (14.55)
LDL During Second Trimester, mg/dL (*M*, *SD*)	360	125.33 (34.65)
Triglycerides During Second Trimester, mg/dL (*M*, *SD*)	360	133.64 (64.04)
Glucose During Second Trimester, mg/dL (*M*, *SD*)	360	79.49 (10.05)
Insulin During Second Trimester, pmol/L (*M*, *SD*)	359	79.41 (108.29)
C-peptide During Second Trimester, nmol/L (*M*, *SD*)	360	0.66 (0.43)
IL-6 During Third Trimester, pg/mL (*M*, *SD*)	350	0.59 (1.02)
TNF-a During Third Trimester, pg/mL (*M*, *SD*)	350	5.81 (1.84)
CRP During Third Trimester, mg/L (*M*, *SD*)	350	5.11 (4.30)
**Infant**		
Weight-for-length (*M*, *SD*)		
At birth	331	-0.44 (1.51)
At 2 months	331	-0.40 (1.52)
At 6 months	293	-0.06 (1.15)
At 1 year	298	0.30 (0.97)

*Notes:* Untransformed data are presented. *CRP* C-Reactive Protein, *IL-6* Interleukin 6, *TNF-a* Tumor Necrosis Factor Alpha

Estimates for associations of maternal ultra-processed food intake with maternal weight change and cardiometabolic markers are presented in Tables 2 and 3, respectively. Holding covariates and total energy intake constant, a 1-SD greater percent daily energy intake from ultra-processed foods during pregnancy was associated with 31% higher odds of excessive gestational weight gain and 0.68±0.29 mg/L higher CRP during pregnancy. Holding covariates and total energy intake constant, a 1-SD greater percent daily energy intake from ultra-processed foods during pregnancy was associated with 6.7±3.4% greater gestational weight gain retained and 1.09±0.36 kg greater postpartum weight retention; however, ultra-processed food intake during postpartum was not significantly associated with these weight outcomes. Associations of maternal ultra-processed food intake with other maternal weight change and cardiometabolic marker variables were non-significant.

**Table 2:** Estimates for associations of maternal ultra-processed food intake with maternal weight change

	**%Energy Intake from Ultra-Processed Foods (z-scored)**					
	**During Pregnancy**			**During Postpartum**		
	**OR**	***p***	**95% CI**	**OR**	***p***	**95% CI**
Gestational Weight Gain						
Model 1						
Inadequate > Adequate	0.88	.461	0.64, 1.23	--	--	--
Excessive > Adequate	1.33	.033	1.02, 1.73	--	--	--
Model 2^a^						
Inadequate > Adequate	0.88	.451	0.63, 1.23	--	--	--
Excessive > Adequate	1.31	.045	1.01, 1.70	--	--	--
	***B±SE***	***p***	**95% CI**	***B±SE***	***p***	**95% CI**
Gestational Fat Gain (kg)						
Model 1	0.07±0.20	.712	-0.32, 0.46	--	--	--
Model 2^a,b,c,d^	0.32±0.19	.094	-0.05, 0.69	--	--	--
%Gestational Weight Gain Retained						
Model 1	7.68±3.29	.020	1.20, 14.17	-3.81±3.35	.256	-10.41, 2.79
Model 2^a,c,d^	6.66±3.37	.049	0.03, 13.30	-4.33±3.39	.202	-11.00, 2.34
Postpartum Weight Change (kg)						
Model 1	1.19±0.35	<.001	0.50, 1.87	-0.36±0.36	.318	-1.06, 0.35
Model 2^a,c,d^	1.09±0.36	.003	0.38, 1.80	-0.44±0.36	.222	-1.14, 0.27

*Notes:* In pregnancy and postpartum models, adjusted for ^a^total energy intake. In pregnancy models, adjusted for ^b^early pregnancy body mass index, ^c^age, ^d^income-poverty ratio

**Table 3:** Estimates for associations of maternal ultra-processed food Intake with cardiometabolic markers obtained during the second and third trimesters

	%Energy Intake from Ultra-Processed Foods During Pregnancy (z-scored)		
	*B±SE*	*p*	95% CI
Second Trimester			
HDL (mg/dL)			
Model 1	-0.95±0.80	.232	-2.52, 0.61
Model 2^a,b,c^	-0.14±0.96	.885	-2.03, 1.76
LDL (mg/dL)			
Model 1	-0.50±1.90	.791	-4.25, 3.24
Model 2^a^	-0.73±1.92	.705	-4.49, 3.04
Triglycerides (mg/dL)			
Model 1	4.11±3.59	.254	-2.95, 11.16
Model 2^a,b,c,d^	-0.89±3.18	.779	-7.14, 5.37
Glucose (mg/dL)			
Model 1	-0.70±0.48	.145	-1.65, 0.24
Model 2^a,d^	-0.39±0.49	.425	-1.35, 0.57
Insulin (pmol/L)			
Model 1	5.00±4.23	.238	-3.32, 13.31
Model 2^a,b,c^	2.27±4.92	.645	-7.43, 11.96
C-peptide (nmol/L)			
Model 1	0.04±0.02	.066	0.00, 0.08
Model 2^a,b,c^	0.03±0.02	.247	-0.02, 0.08
Third Trimester			
IL-6 (pg/mL)			
Model 1	0.07±0.06	.277	-0.05, 0.19
Model 2^a,b^	0.06±0.07	.346	-0.07, 0.19
TNF-a (pg/mL)			
Model 1	0.18±0.11	.090	-0.03, 0.39
Model 2^a,b^	0.10±0.11	.356	-0.12, 0.33
CRP (mg/L)			
Model 1	0.89±0.25	<.001	0.39, 1.39
Model 2^a,b,c,d,e,f^	0.68±0.29	.021	0.10, 1.26

*Notes:CRP* C-Reactive Protein, *IL-6* Interleukin 6, *TNF-a* Tumor Necrosis Factor Alpha. Adjusted for ^a^total energy intake, ^b^income-poverty ratio, ^c^moderate- and vigorous-intensity physical activity, ^d^age, ^e^low-intensity physical activity, and ^f^early pregnancy body mass index

Estimates for associations of maternal ultra-processed food intake with infant weight-for-length are presented in Table 4. On average, infant weight-for-length z-score at birth (intercept) was -0.50*±*0.07 (*p* < .001, 95% CI [-0.64, -0.35]) and infant weight-for-length z-score trajectory from birth to 1 year (slope) was 0.75*±*0.10 (*p* < .001, 95% CI [0.56, 0.95]). Positive associations of maternal ultra-processed food intake during pregnancy with infant weight-for-length at birth and with the trajectory from birth to 1 year were non-significant. The negative association of maternal ultra-processed food intake during postpartum with infant weight-for-length trajectory from birth to 1 year was also non-significant.

**Table 4:** Estimates for associations of maternal ultra-processed food intake with infant weight-for-length

	%Energy Intake from Ultra-Processed Foods (z-scored)					
	During Pregnancy			During Postpartum		
	*γ±SE*	*p*	95% CI	*γ±SE*	*p*	95% CI
Weight-for-length at birth (Intercept)						
Model 1	0.02±0.08	.785	-0.14, 0.19	--	--	--
Model 2^a^	0.02±0.08	.817	-0.15, 0.18	--	--	--
Weight-for-length trajectory from birth to 1 year (Slope)						
Model 1	0.10±0.12	.405	-0.13, 0.33	-0.04±0.08	.610	-0.19, 0.11
Model 2^a^	0.11±0.12	.361	-0.12, 0.34	-0.06±0.08	.472	-0.21, 0.10

*Notes:* In pregnancy and postpartum models, adjusted for ^a^total energy intake

## Discussion

Greater ultra-processed food intake during pregnancy was associated with greater gestational weight gain and retention, postpartum weight retention, and pregnancy CRP in a US cohort. However, pregnancy intake of ultra-processed foods was not significantly associated with other indicators of maternal cardiometabolic health and infant weight-for-length, and postpartum intake was not significantly associated with maternal or infant outcomes. The magnitude and significance pattern of the results were consistent when controlling for total energy intake.

Women consumed just over half their daily energy intake from ultra-processed foods during pregnancy and postpartum, consistent with observations in non-pregnant U.S. populations [1]. The positive relation of ultra-processed food intake during pregnancy with gestational weight gain corroborates with previous findings [6, 7, 11], while the positive relations with gestational weight gain retained and postpartum weight retention were previously unexplored. Prior work showed a higher pro-inflammatory potential of diet for women who consumed more ultra-processed foods during pregnancy [8], which the present study also built upon by finding a positive association of maternal ultra-processed food intake with CRP during pregnancy. However, the lack of significant relations of pregnancy ultra-processed food intake with pregnancy elevated glucose and infant weight-for-length is inconsistent with prior studies [6, 10, 11]. Prior studies included smaller sample sizes [6, 11], samples with pregestational diabetes [6], dietary assessment using food frequency questionnaires [6, 10, 11], adjustment for different covariates (e.g., number of prenatal consultations) [6, 10], and different infant weight and adiposity measurements (i.e., time to infant overweight, infant percent body fat) [10, 11], which may explain inconsistent findings.

The present study findings do not elucidate the mechanism(s) by which pregnancy intake of ultra-processed foods would promote adverse maternal weight outcomes and inflammation. Future research investigating mechanisms, including increased total energy and added sugars intake [17, 33], displacement of nutrient-rich foods [34], protein leverage (i.e., overconsuming low-protein foods to meet protein needs) [33, 35], quicker eating rate [33], exposure to phthalates and bisphenol [21], and alterations to the gut microbiome [36], is warranted. In the present study, positive associations of ultra-processed food intake with maternal weight gain and retention and pregnancy CRP did not weaken when statistically accounting for total energy intake, suggesting that increased total energy may not be a primary mechanism.

Strengths and limitations of the present study should be considered when interpreting findings. This secondary analysis was strengthened by the relatively large sample size wherein over half of the women were with overweight or obesity, enhancing internal validity. Internal validity was further strengthened by the prospective study design; repeated measures of maternal diet and anthropometrics throughout pregnancy and postpartum and infant anthropometrics from birth to age 1 year; directly measured maternal and infant weight; multiple maternal cardiometabolic biomarkers; and measurement of and adjustment for several confounds including maternal physical activity and total energy intake.

Ultra-processed food intake was assessed according to the NOVA classification system by using data from multiple 24-hour dietary recalls, the least biased self-report assessment available [37]. Although misclassification is possible when applying NOVA [38], the potential for misclassification was mitigated by using standardized statistical code for applying NOVA and by disaggregating foods to underlying ingredients [17]. Limitations of NOVA, however, are that the categories lack quantitative cutoffs (e.g., two foods could both be in group 3 regardless of differing amounts of added processed culinary ingredients), that food additives may be legally permitted in a food but not present, and that nutritious and sustainable foods (e.g., soy-based meat and dairy alternatives) can be classified as ultra-processed [39, 40].

Given the observational study design, causal inferences from the present study are limited. The temporal precedence of ultra-processed food intake was limited in some of the examined associations. Specifically, dietary assessments were averaged across pregnancy to examine associations with cardiometabolic markers obtained at the second and third trimesters, and dietary assessments were averaged across postpartum to examine the association with infant weight-for-length slope from birth to 1 year. However, averaging of multiple dietary recalls provides a more valid estimate of typical intake than does a single recall, and dietary recalls, cardiometabolic markers, and weight are thought to reflect individuals’ typical behaviors and health status. The sociodemographic characteristics were consistent with those from the metropolitan area in North Carolina, US, but are not representative of women throughout the US.

Limitations notwithstanding, the current study findings support interventions that reduce exposure to ultra-processed foods in pregnant women. Public health proposals include strategies to de-incentivize ultra-processed food intake, such as changing regulations for ultra-processed food production, increasing taxation of ultra-processed foods, and decreasing subsidies for ultra-processed food ingredients [41]. Proposed strategies to incentivize unprocessed and minimally processed food intake include increasing accessibility to and affordability of meal kits and meal sharing [41]. In a recent non-randomized trial in Brazilian women, an educational intervention during routine prenatal medical visits reduced maternal ultra-processed food intake by 19% [42], demonstrating the efficacy of targeting this behavioral risk factor at the individual level. Randomized controlled trials in diverse populations are also needed to test whether targeting ultra-processed food intake during pregnancy may support optimal maternal weight change and health.

## Conclusions

Findings indicate that maternal pregnancy intake of ultra-processed foods may be a modifiable behavioral risk factor for excessive maternal weight change and inflammation for women in the US. Although the present study findings need to be confirmed in other pregnant populations, decreasing maternal ultra-processed food intake may be an important behavioral target in prenatal interventions.

## Supplementary Information

**Additional file 1.**

**Additional file 2.**

**Additional file 3.**

## Data Availability

Data described in the manuscript, code book, and analytic code will be made available from the corresponding author upon request pending application and approval.

## Abbreviations

ASA24: Automated Self-Administered 24-Hour Dietary Recall
BMI: Body Mass Index
CRP: C-Reactive Protein
FNDDS: Food and Nutrient Database for Dietary Studies
IL-6: Interleukin 6
PEAS: Pregnancy Eating Attributes Study
REML: Restricted Maximum Likelihood Estimation,
TNF-a: Tumor Necrosis Factor Alpha
