# Supplementary material for: Associations of ultra-processed food intake with maternal weight change and cardiometabolic health and infant growth
Source: Int J Behav Nutr Phys Act. 2022 May 26;19:61. doi: 10.1186/s12966-022-01298-w (PMC9137185; doi:10.1186/s12966-022-01298-w)
Supplement: Supplementary file 1 — Additional file 1. [file 12966_2022_1298_MOESM1_ESM.docx]

Additional File 1

*15 Most-Frequently Reported Foods from NOVA Ultra-Processed Food Category*

| **Pregnancy** | **Postpartum** |
| --- | --- |
| *FNDDS Food or ingredient description* | *FNDDS Food or ingredient description* |
| 1. Tomato catsup (ketchup) | 1. Muffin, pumpkin |
| 2. Mayonnaise, regular | 2. Bread, fruit, without nuts |
| 3. Macaroni or noodles with cheese, from boxed mix with already prepared cheese sauce | 3. Chocolate, sweet or dark |
| 4. Bread, fruit, without nuts | 4. Italian dressing, made with vinegar and oil |
| 5. Cheese, processed, American or Cheddar | 5. Mayonnaise, regular |
| 6. Fruit flavored drink | 6. Tea, leaf, presweetened with sugar |
| 7. Yogurt, fruit variety, low-fat milk | 7. Salty snacks, corn or cornmeal base, tortilla chips |
| 8. Italian dressing, made with vinegar and oil | 8. Tomato catsup (ketchup) |
| 9. Roll, white, soft | 9. Soft drink, cola-type, sugar-free |
| 10. Cookie, chocolate chip, made from home recipe or purchased at a bakery | 10. Cookie, chocolate chip, made from home recipe or purchased at a bakery |
| 11. Muffin, pumpkin | 11. Cream substitute, flavored, liquid |
| 12. White potato, French fries, from frozen, deep fried | 12. Yogurt, fruit variety, low-fat milk |
| 13. Salsa, red, cooked, not homemade | 13. Bread, whole wheat, NS as to 100% |
| 14. White potato, chips | 14. Roll, white, soft |
| 15. Bread, white | 15. Bread, pumpkin |
