# Supplementary material for: Associations of ultra-processed food intake with maternal weight change and cardiometabolic health and infant growth
Source: Int J Behav Nutr Phys Act. 2022 May 26;19:61. doi: 10.1186/s12966-022-01298-w (PMC9137185; doi:10.1186/s12966-022-01298-w)
Supplement: Supplementary file 2 — Additional file 2. [file 12966_2022_1298_MOESM2_ESM.docx]

Additional File 2

*Bivariate Correlations Among Continuous Variables*

|  | 1. | 2. | 3. | 4. | 5. | 6. | 7. | 8. | 9. | 10. | 11. | 12. | 13. | 14. | 15. | 16. | 17. | 18. | 19. | 20. | 21. |
| --- | --- | --- | --- | --- | --- | --- | --- | --- | --- | --- | --- | --- | --- | --- | --- | --- | --- | --- | --- | --- | --- |
| **Maternal** |  |  |  |  |  |  |  |  |  |  |  |  |  |  |  |  |  |  |  |  |  |
| 1. Age | -- | .03 | -.01 | .34*** | -.09 | -.22*** | .10 | -.08 | -.03 | .16** | -.13* | -.16** | .02 | .07 | .11* | .15** | -.07 | -.08 | -.04 | -.19*** | -.13* |
| 2. LI Physical Activity |  | -- | .23*** | .09 | -.05 | -.17** | -.08 | -.14* | -.11 | .02 | -.06 | -.08 | .07 | -.05 | -.04 | -.03 | -.10 | -.10 | -.08 | -.01 | -.15* |
| 3. MVI Physical Activity |  |  | -- | .14* | -.07 | -.12* | .01 | -.10 | -.14* | .03 | -.13 | -.10 | .16* | .00 | -.15* | -.08 | -.15* | -.19** | -.03 | .09 | -.16** |
| 4. Income-Poverty Ratio |  |  |  | -- | -.10 | -.15** | -.07 | -.12 | -.31*** | .24*** | -.14* | -.19** | .22*** | .08 | -.11* | -.08 | -.27*** | -.29*** | -.15** | -.16** | -.21*** |
| 5. Total Energy Intake – Preg |  |  |  |  | -- | .11* | .37*** | .04 | .05 | .01 | .04 | -.03 | -.17** | .05 | -.01 | -.01 | .04 | .04 | .05 | .12* | .01 |
| 6. %Energy Intake from UPF – Preg |  |  |  |  |  | -- | .00 | .39*** | .14** | .02 | .21*** | .14* | -.07 | -.01 | .06 | -.08 | .07 | .10 | .06 | .10 | .19*** |
| 7. Total Energy Intake – Post |  |  |  |  |  |  | -- | .13* | .08 | -.06 | .10 | .05 | -.18** | .06 | .05 | .04 | .00 | .02 | .05 | .13* | .01 |
| 8. %Energy Intake from UPF – Post |  |  |  |  |  |  |  | -- | .09 | -.09 | -.06 | -.07 | -.08 | .00 | .15* | .11 | .16* | .14* | -.08 | .11 | .06 |
| 9. Early Preg BMI |  |  |  |  |  |  |  |  | -- | -.41*** | .09 | .11 | -.33*** | .00 | .14** | .40*** | .51*** | .55*** | .15** | .13* | .40*** |
| 10. Gestational Fat Gain |  |  |  |  |  |  |  |  |  | -- | .17** | .07 | .23*** | .03 | -.17** | -.19*** | -.27*** | -.30*** | -.09 | -.12* | -.18*** |
| 11. Postpartum Weight Change |  |  |  |  |  |  |  |  |  |  | -- | .88*** | -.11 | -.03 | -.05 | -.08 | .02 | .02 | .21*** | .11 | .10 |
| 12. %GWG Retained |  |  |  |  |  |  |  |  |  |  |  | -- | -.13* | -.03 | -.10 | -.07 | .02 | .00 | .22*** | .12* | .09 |
| 13. HDL |  |  |  |  |  |  |  |  |  |  |  |  | -- | .05 | -.34*** | -.23*** | -.27*** | -.30*** | -.08 | -.18*** | -.13* |
| 14. LDL |  |  |  |  |  |  |  |  |  |  |  |  |  | -- | .02 | -.04 | -.05 | -.02 | .02 | -.01 | .04 |
| 15. Triglycerides |  |  |  |  |  |  |  |  |  |  |  |  |  |  | -- | .18*** | .21*** | .28*** | -.03 | .04 | .11 |
| 16. Glucose |  |  |  |  |  |  |  |  |  |  |  |  |  |  |  | -- | .64*** | .62*** | .08 | .04 | .03 |
| 17. Insulin |  |  |  |  |  |  |  |  |  |  |  |  |  |  |  |  | -- | .91*** | .14* | .14** | .17** |
| 18. C-peptide |  |  |  |  |  |  |  |  |  |  |  |  |  |  |  |  |  | -- | .12* | .16** | .21*** |
| 19. IL-6 |  |  |  |  |  |  |  |  |  |  |  |  |  |  |  |  |  |  | -- | .10 | .08 |
| 20. TNF-a |  |  |  |  |  |  |  |  |  |  |  |  |  |  |  |  |  |  |  | -- | .16** |
| 21. CRP |  |  |  |  |  |  |  |  |  |  |  |  |  |  |  |  |  |  |  |  | -- |

*Notes:* LI = Low-Intensity, MVI = Moderate- and Vigorous-Intensity, Preg = Pregnancy, Post = Postpartum, UPF = Ultra-Processed Food, BMI = Body Mass Index, GWG = Gestational Weight Gain, IL-6 = Interleukin 6, TNF-a = Tumor Necrosis Factor Alpha, CRP = C-Reactive Protein.
