# Supplementary material for: Associations of ultra-processed food intake with maternal weight change and cardiometabolic health and infant growth
Source: Int J Behav Nutr Phys Act. 2022 May 26;19:61. doi: 10.1186/s12966-022-01298-w (PMC9137185; doi:10.1186/s12966-022-01298-w)
Supplement: Supplementary file 3 — Additional file 3. [file 12966_2022_1298_MOESM3_ESM.docx]

Additional File 3

*Means and Standard Deviations of Continuous Variables by Categorical Variables and ANOVA estimates*

|  | Maternal Smoking Status | | | | Maternal Alcohol Use During Pregnancy | | | Maternal Gestational Weight Gain | | | |
| --- | --- | --- | --- | --- | --- | --- | --- | --- | --- | --- | --- |
|  | Smoker During Pregnancy | Former Smoker | Never Smoker | *F* | No | Yes | *F* | Inadequate | Adequate | Excessive | *F* |
| **Maternal** |  |  |  |  |  |  |  |  |  |  |  |
| Smoking Status | -- | -- | -- | -- | 2.82 (0.43) | 2.71 (0.52) | 3.83 | 2.76 (0.47) | 2.84 (0.46) | 2.73 (0.48) | 1.55 |
| Alcohol Use During Pregnancy | 0.57 (0.53) | 0.55 (0.50) | 0.41 (0.49) | 2.04 | -- | -- | -- | 0.40 (0.49) | 0.41 (0.49) | 0.47 (0.50) | 0.60 |
| Gestational Weight Gain | 2.14 (0.69) | 2.45 (0.81) | 2.27 (0.74) | 1.32 | 2.27 (0.75) | 2.36 (0.74) | 1.01 | -- | -- | -- | -- |
| Age | 25.00 (3.42) | 30.49 (4.61) | 30.86 (4.56) | 5.69** | 30.70 (4.45) | 30.60 (4.86) | 0.03 | 30.90 (4.42) | 30.36 (4.50) | 30.66 (4.72) | 0.33 |
| LI Physical Activity | 4.25 (5.32) | 2.78 (3.18) | 4.35 (4.46) | 2.04 | 4.25 (4.14) | 3.88 (4.54) | 0.41 | 3.48 (3.29) | 4.51 (5.23) | 3.39 (3.58) | 2.13 |
| MVI Physical Activity | 1.00 (1.41) | 0.81 (1.41) | 1.15 (2.14) | 0.43 | 0.89 (2.11) | 1.32 (1.90) | 2.54 | 1.26 (2.16) | 1.12 (2.36) | 0.97 (1.75) | 0.39 |
| Income-Poverty Ratio | 1.53 (1.95) | 3.06 (1.90) | 4.20 (1.81) | 13.66*** | 3.77 (2.00) | 4.13 (1.81) | 2.34 | 3.40 (2.16) | 3.89 (1.87) | 3.99 (1.95) | 2.11 |
| Total Energy Intake – Preg | 2055.52 (614.03) | 2284.00 (880.95) | 1947.32 (610.04) | 4.95** | 2022.53 (745.29) | 1998.96 (589.23) | 0.08 | 1976.52 (722.33) | 1966.12 (600.17) | 2089.10 (734.13) | 1.24 |
| %Energy Intake from UPF – Preg | 0.60 (0.13) | 0.53 (0.14) | 0.52 (0.14) | 0.83 | 0.52 (0.15) | 0.53 (0.13) | 0.53 | 0.49 (0.17) | 0.51 (0.14) | 0.55 (0.13) | 4.15* |
| Total Energy Intake – Post | 2326.62 (655.16) | 1974.29 (634.28) | 1940.85 (635.83) | 0.57 | 1929.94 (683.80) | 1977.89 (566.88) | 0.30 | 2066.27 (668.63) | 1876.12 (559.61) | 2015.61 (703.09) | 1.72 |
| %Energy Intake from UPF – Post | 0.61 (0.32) | 0.53 (0.16) | 0.49 (0.16) | 1.39 | 0.52 (0.18) | 0.48 (0.14) | 3.20 | 0.50 (0.17) | 0.51 (0.18) | 0.51 (0.16) | 0.01 |
| Early Preg BMI | 28.93 (6.75) | 29.57 (5.70) | 26.24 (6.77) | 5.66** | 26.67 (6.91) | 27.23 (6.42) | 0.49 | 26.90 (7.34) | 25.33 (6.54) | 28.03 (5.88) | 6.38** |
| Gestational Fat Gain | -0.78 (2.55) | -0.14 (4.00) | 0.82 (3.28) | 2.18 | 0.78 (3.49) | 0.42 (3.34) | 0.73 | -2.83 (3.03) | 0.06 (2.59) | 2.13 (3.10) | 69.82*** |
| Postpartum Weight Change | 5.53 (0.38) | 0.54 (5.75) | 0.86 (4.75) | 1.44 | 0.63 (4.70) | 1.17 (5.24) | 0.70 | -0.91 (5.36) | 0.03 (4.32) | 2.08 (5.79) | 8.18*** |
| %GWG Retained | 62.58 (29.19) | -6.29 (68.69) | 3.23 (48.18) | 2.58 | -1.15 (49.52) | 6.74 (56.15) | 1.29 | -10.48 (63.41) | -2.98 (54.72) | 13.72 (42.33) | 5.38** |
| HDL | 61.71 (10.64) | 74.70 (16.32) | 73.69 (13.98) | 2.54 | 73.24 (14.45) | 73.81 (14.41) | 0.10 | 71.14 (17.30) | 74.55 (13.94) | 73.54 (13.42) | 1.18 |
| LDL | 110.00 (44.89) | 130.07 (36.40) | 125.58 (34.84) | 1.04 | 123.71 (35.32) | 128.87 (35.50) | 1.41 | 125.26 (46.33) | 124.27 (32.38) | 127.55 (31.05) | 0.32 |
| Triglycerides | 167.71 (51.40) | 131.21 (44.71) | 129.45 (47.24) | 2.26 | 133.03 (52.20) | 128.06 (39.86) | 0.74 | 152.58 (114.41) | 130.22 (42.12) | 127.29 (46.07) | 3.82* |
| Glucose | 86.00 (20.46) | 81.65 (9.34) | 78.55 (9.45) | 3.58* | 79.66 (11.47) | 78.81 (7.49) | 0.49 | 80.06 (13.34) | 78.42 (11.28) | 79.83 (7.07) | 0.85 |
| Insulin | 181.43 (221.13) | 94.98 (84.86) | 69.36 (109.15) | 4.35* | 75.97 (130.03) | 77.75 (80.15) | 0.02 | 85.08 (90.53) | 77.47 (153.16) | 75.21 (70.72) | 0.19 |
| C-peptide | 1.15 (1.07) | 0.73 (0.46) | 0.60 (0.37) | 7.14** | 0.64 (0.43) | 0.65 (0.42) | 0.05 | 0.69 (0.47) | 0.62 (0.49) | 0.64 (0.34) | 0.71 |
| IL-6 | 0.42 (0.20) | 0.52 (0.42) | 0.66 (1.24) | 0.46 | 0.61 (0.80) | 0.65 (1.43) | 0.09 | 0.54 (0.53) | 0.56 (0.67) | 0.63 (1.32) | 0.28 |
| TNF-a | 7.13 (1.76) | 5.88 (1.82) | 5.82 (1.90) | 1.65 | 5.86 (2.00) | 5.86 (1.76) | 0.00 | 5.95 (1.90) | 5.67 (2.04) | 5.83 (1.69) | 0.49 |
| CRP | 9.19 (11.31) | 4.65 (4.06) | 5.26 (4.09) | 3.28* | 5.13 (4.09) | 5.45 (4.80) | 0.35 | 4.77 (3.97) | 5.21 (4.91) | 5.22 (4.03) | 0.28 |

*Notes:* Untransformed data are presented. LI = Low-Intensity, MVI = Moderate- and Vigorous-Intensity, Preg = Pregnancy, Post = Postpartum, UPF = Ultra-Processed Food, BMI = Body Mass Index, GWG = Gestational Weight Gain, IL-6 = Interleukin 6, TNF-a = Tumor Necrosis Factor Alpha, CRP = C-Reactive Protein. **p* < .05, ***p* <.01, ****p* < .001
